# The COVID-19 Pandemic as an Impulse for the Development of Telemedicine in Primary Care in Poland

**DOI:** 10.3390/jpm12071165

**Published:** 2022-07-18

**Authors:** Kamila Furlepa, Andrzej Śliwczyński, Karolina Kamecka, Remigiusz Kozłowski, Izabela Gołębiak, Dominika Cichońska-Rzeźnicka, Michał Marczak, Wojciech Michał Glinkowski

**Affiliations:** 1 Satellite Campus in Warsaw, University of Humanities and Economics in Lodz, 90-212 Lodz, Poland; 2 Department of Management and Logistics in Healthcare, Medical University of Lodz, 90-131 Lodz, Poland; kkamecka@gmail.com (K.K.); michal.marczak@umed.lodz.pl (M.M.); 3 Center of Security Technologies in Logistics, Faculty of Management, University of Lodz, 90-237 Lodz, Poland; remigiusz.kozlowski@wz.uni.lodz.pl; 4 Lazarski University, 02-662 Warsaw, Poland; izabela.golebiak@lazarski.pl; 5 Department of Social Medicine, Medical University of Lodz, 90-131 Lodz, Poland; dominika.cichonska@umed.lodz.pl; 6 Center of Excellence “TeleOrto” for Telediagnostics and Treatment of Disorders and Injuries of the Locomotor System, Department of Medical Informatics and Telemedicine, Medical University of Warsaw, 00-581 Warsaw, Poland; w.glinkowski@gmail.com; 7 Polish Telemedicine and eHealth Society, 03-728 Warsaw, Poland

**Keywords:** telemedicine, patient, virtual primary care, teleconsultation, COVID-19

## Abstract

Telemedicine gives a safe and effective way of providing healthcare. During the COVID-19 pandemic, it was possible to offer teleconsultations in primary care (Primary Care Teleconsultation-PCT). The study aimed to present an analysis of the PCTs served in the years 2020–2021 in the field of primary care in Poland to determine how the COVID-19 pandemic contributed to the development of telemedicine in primary care in Poland. The database, containing a list of medical services provided remotely obtained from the National Health Fund, was analyzed. Economic and tax indicators obtained from the Ministry of Finance were also analyzed. Personal Income Tax (PIT) value was used as an indicator of household wealth, and the Corporate Income Tax (CIT) was used as an indicator of economic activity in individual counties for 2019. Along with the COVID-19 pandemic, patients as healthcare beneficiaries can take advantage of previously unserved telemedicine services as part of primary care. The data analysis showed that, along with the introduced recommendations and restrictions in connection with the pandemic, the number of teleconsultations in 2021 increased compared to 2020. In response to the pandemic, an educational campaign targeted older patients. These indicate the most significant percentage of PCTs among patients aged 70 and older. The study shows that the awareness barrier in implementing services for the elderly population decreased significantly. There was a clear correlation between the increase in PCTs and patient age.

## 1. Introduction

Recently, telemedicine (TM) has been widely accepted as a safe and effective way to deliver healthcare [1,2,3], used by physicians of many specialties. In principle, it should be assumed that telemedicine means ‘healing at a distance’, which was first coined in the 1970s [4,5], which entails using information and communication technologies to improve patient outcomes by increasing access to care and medical information. Telemedicine, as described by the WHO, refers to: “The delivery of healthcare services, where distance is a critical factor, by all healthcare professionals using information and communication technologies for the exchange of valid information for the diagnosis, treatment, and prevention of disease and injuries, research and evaluation, and for the continuing education of healthcare providers, all in the interests of advancing the health of individuals and communities” [6]. According to the approach recommended by the Polish Telemedicine and eHealth Society, the term “telemedicine” refers to healthcare services, along with professional and legal liability [7] on equal terms with a personally provided healthcare service. The described approach has been legally sanctioned in Poland [8]. The COVID-19 pandemic has significantly intensified the transformation of telemedicine since the beginning of 2020 and accelerated the transition to remote consultations (patient/physician teleconsultations and physician/physician teleconsultations) in medicine [9,10,11,12,13,14,15,16]. Lockdown has accelerated the implementation of TM in most countries around the world [3,17,18,19]. The role of TM is rapidly evolving across medical specialties [9,10,11,12,13,14,15,16,17,18,19,20,21]. Teleconsultations and remote examinations of patients have become part of everyday medical practice and have become an experience familiar to patients [22].

In Poland, it has been possible since 2015 to legally provide remote healthcare services, based on Article 3, paragraph 1 of the Act on Medical Activity, which stipulates that “Medical activity consists in delivering medical services. These services may be provided through ICT(Information and Communication Technology) or communication systems [23]. Due to the SARS-CoV-2 pandemic, reimbursable teleconsultation can be provided as part of primary care from August 2020” [24]. The rapid adoption of telemedicine in various specialties and countries has shown promising results, with high satisfaction indices recorded [25], due to the ability to provide high-quality care to patients with a minimized risk of disease transmission.

Primary care is defined as a fundamental and universal healthcare system unit based on practice, science, and acceptable methods. It provides universal access for all citizens and their full participation in close relation to a given country’s financial capacity [26]. Medical teleconsultations are carried out through an ICT system or communication system, which in practice means mainly the use of mobile phones and the Internet (they can also take the form of text, verbal, and image transmission) [14,27]. In Poland, it is broadly understood that telemedicine services are a tool of consultation between healthcare professionals and patients, provided on many levels, but also a tool of consultation between healthcare professionals, such as physicians, nurses, or midwives.

Many barriers were indicated in the analysis conducted before the COVID-19 pandemic concerning telemedicine services [28]. In many countries, the COVID-19 pandemic contributed to increased tele-visits provided in primary care. Similarly, in Poland, with the first cases of COVID-19 infection, physicians began providing tele-visits as part of primary care [29].

This study aims to present an analysis of primary care teleconsultations served in the years 2020–2021 in Poland, in the context of determining how the COVID-19 pandemic contributed to the development of telemedicine in primary care in Poland.

## 2. Materials and Methods

A database, containing a list of healthcare services provided remotely, was obtained from the National Health Fund (NFZ), the only public payer for healthcare services in Poland. All of the available data on PCTs in Poland were used for research purposes, in the form of tables and graphs. The tables used a color scale to visualize the differences better. The color intensity selection mechanism in the Excel spreadsheet was used, in which the red color means the highest value in a given set, and the green color is the lowest. The database contained information collected in the years 2020–2021, with the reservation that the data for 2021 may be slightly adjusted in 2022 due to settling healthcare services, which ends in April–May 2022. The data for 2019 were not included in the analysis, due to the course of the pandemic in Poland compared to other countries, as the first case of SARS-CoV-2 in Poland was diagnosed in March 2020. As a result of the rapid spread of the pandemic, the teleconsultations within primary care began [29]. Economic data were obtained from the Ministry of Finance. The Personal Income Tax (PIT) value was used as an indicator of household wealth. The Corporate Income Tax (CIT) was used to indicate economic activity in individual counties for 2019. The mean values, medians, and Pearson’s correlation coefficients were calculated for the voivodeships. The relationships were calculated for pairs of variables: average and median PIT and CIT values and the frequency of teleconsultations per 10,000 residents [14].

The calculations were carried out for the economic and tax data for 2019, to eliminate the impact of lockdowns in 2020 and 2021. The data derived from the Ministry of Finance were aggregated at the voivodeship level. The relationship between the number of patients who used the PCT, with average and median values per county in an individual voivodeship, was examined. When giving a teleconsultation, a physician is entitled to issue prescriptions, sick leave notes, referrals, or orders for additional tests or healthcare services. The course of a medical teleconsultation (virtual visit) is documented in the patient’s electronic medical record [14].

The patient data were aggregated and not analyzed individually, which allowed for compliance with the condition of protecting sensitive personal data. The analysis was completed with a breakdown by age presented in 5-year age groups, in line with the groups reported in the official statistics of Statistics Poland (GUS-chief government executive agency charged with collecting and publishing statistics related to the country’s economy, population, and society, at the national and local levels). Due to the necessity of making comparisons between the voivodeships, the analysis results were presented based on the population of a given voivodeship, according to Statistics Poland. The data for the statistical calculations were prepared in a spreadsheet. The statistical analysis was performed using the TIBCO Statistica^®^ data analysis software system, version 13. (TIBCO Software Inc. 2017—Tulsa, OK, USA).

## 3. Results

Although physicians have been able to offer teleconsultations to patients through ICT and communication systems since 2015, the use of such opportunities has been infrequent. In the years 2020–2021, the number of PCTs increased. Table 1 presents the incidence of PCT per 10,000 residents.

**Figure 1:**
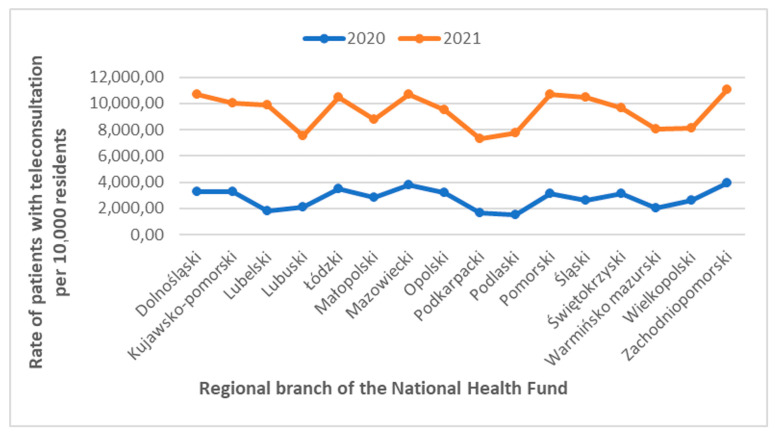
The diagram shows the growth of the primary care teleconsultations in voivodeships in 2020 and 2021.

The highly urbanized Zachodniopomorskie and Mazowieckie voivodeships dominated in both years, with many PCTs. In the following 2021, some of the voivodeships achieved indicators exceeding 10,000. These were the following voivodeships: Zachodniopomorskie, Mazowieckie, Pomorskie, Dolnośląskie, Łódzkie, and Śląskie. The highest increase in the primary care teleconsultations (PCT) ratio per 10,000 inhabitants was recorded in the Lubelskie and Podlaskie voivodeships. The incidence of PCTs provided in these two voivodeships increased over five times. The PCT incidence decrease was not observed in any of the voivodeships (Table 1, Figure 1).

Figure 1 shows the incidence growth of the number of PCTs in the individual National Health Fund regional branches. In 2021, the lowest rate recorded in the Podkarpackie voivodeship was over 1.8 times higher than the highest rate in 2020 (Zachodniopomorskie voivodeship).

**Figure 2:**
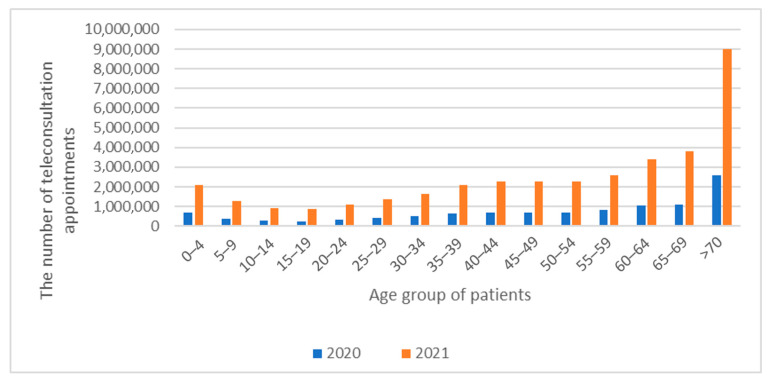
The distribution diagram of the served PCTs in 2020–2021, in age groups.

The distribution of the PCTs served, in age groups, is presented in Figure 2.

Among the patients receiving primary care teleconsultations in 2020 and 2021, the most numerous group were people aged 70 and older (24% of all of the PCTs). It should be remembered that Poland’s expected average survival age in 2020 was 77 years [30].

Table 2 shows the relationship between the age groups and the provided PCTs. The color scale visualizes the position of a number in a series of values, from red being the highest value to green being the lowest value. There was an increase in the percentage of the PCTs served in 2020 and 2021. The rate of the PCTs given increases with the age, starting from the 15–19 age group. However, this dependence does not apply to the 0–14 age group.

Table 2 and Table 3 use a color scale to show the lowest, highest, and intermediate values. Pearson’s correlation coefficients were calculated for the indicators of patients who received a PCT per 10,000 inhabitants, according to the average and median values of PIT and CIT in individual voivodeships. The average amount of PIT in 2019 for the percentage of patients who received a PCT in 2020 was 0.47; in 2021—0.57. The average amount of CIT in 2019 for the percentage of patients who received a PCT in 2020 was 0.311; in 2021—0.248, and the median in 2020, respectively, 0.013; and in 2021—0.081. According to the description of Pearson’s correlation function, a weak negative correlation ranges from −0.5 to 0.0, a weak positive correlation ranges from 0.0 to 0.5, a strong negative correlation ranges from −1,0 to −0.5, and a strong positive correlation from 0.5 to 1.0.

In addition, the correlation regarding the average amount of PIT in 2019 with the rate of patients provided with a teleconsultation in 2021 has a value of 0.57, which means a strong positive correlation. Other results indicate a weak positive correlation in the case of the analysis of the average amounts of PIT and CIT. Meanwhile, in the case of the median, which better reflects the finance of the voivodeship population only in the case of the median PIT, the value indicated a weak positive correlation in the case of PIT, while in the case of CIT, the correlation can be considered insignificant.

## 4. Discussion

In the Polish healthcare system, managed by the Ministry of Health and three local government levels, the only public payer for healthcare services is the National Health Fund. Their services are based on 16 regional branches [30,31,32]. The presented results were obtained based on the data on healthcare services financed from the compulsory health insurance. Around 91% of Polish citizens are covered by mandatory insurance, and the remaining element of the uninsured are primarily people living outside the country (who do not pay taxes and contributions related to work in Poland). Under the compulsory health insurance system, people who are not insured are entitled to medical care only in emergencies. Health insurance provides beneficiaries with access to healthcare services that comprise, among others, primary care, outpatient specialist services, and inpatient services. Despite having health insurance, many people decide to obtain subscribed medical services or fully private medical services, paid directly from the patient’s pocket [30].

The right to use primary care services is granted to persons declared on the patient list and persons holding the European Health Insurance Card under the provisions on coordination [33]. The primary care services are provided to a patient by a chosen family physician. The benefits include night and holiday outpatient assistance, visiting medical services, and patient transport [26]. Thanks to this organization, TM made it possible to provide medical care to quarantined patients and offer services to people exposed to infection with a virus of very high virulence. As part of the healthcare services, there are also services performed using telemedicine solutions, based on article 3, paragraph 1 of the Act on Medical Activity, which stipulates the provision of “healthcare services” which may “be provided through ICT or communication systems” [24]. An important issue related to the provision of medical services using telehealth tools is increasing the safety of patients and healthcare personnel [28].

Observing the development of telemedicine in the primary care phenomenon, several phases of PCT implementation in Poland should be indicated. During the pre-pandemic period, very few or no teleconsultation pilots or only experimental services were provided, and reimbursement was not available. During the initial stages of PCT use, a rise in interest among physicians was observed, since the reimbursement value for PCT was officially set at the end of 2019. Initially, there was only an outline of telemedicine use, instead of guidelines regarding the physician’s requirements for providing PCT services to the patient. With a simplification of the technical requirements, PCT consultations were accepted over the phone, as in many countries [34,35,36,37]. The diagnosis verification tools, such as viewing the patient’s medical images, were not systemically implemented. The simplest interview became the primary medical information source noted in the Electronic Health Record. Officially available and analyzed data allow for their presentation in the results subsection. Thus, the compilation of the quantitative results became the basis for analysis and discussion in the proposed study. The qualitative data were not available when conducting the analyses for this study. The financial factors played a role in driving and supporting the adoption of telemedicine solutions. At the beginning of the pandemic period, PCTs were encouraged by the regulations, due to the rising SARS-CoV-2 infection risk [38]. During the midperiod of the pandemic, the situation had changed, and many primary care physicians did not want to return to face-to-face medical services. However, the primary care physicians face-to-face services were encouraged again [33].

According to the analyzed material, there was an apparent increase in the use of teleconsultation with age. Most likely, it is related to the increasing number of diseases occurring with individual age. The adopted PCTs model fully accepted the possibility of teleconsulting primary care over the phone. The analyzed material does not allow for a discussion of the telemedicine tools used during the PCT.

Telehealth combines diagnostics, consultations, treatment, health education, care management, and self-care. The patient participates in telehealth, understood in this context as two-way electronic communication, which brings the patient benefits in the form of time savings and reduces the costs incurred [39]. Telemedicine, a state of providing medical services and healthcare through telecommunications’ tools, is associated with barriers to its adoption. One of the barriers to the implementation of telemedicine services in Poland is the awareness barrier. It applies to supporting medical care for the elderly. There was a noticeable opposition to adopting non-traditional models and the fear that remote research would not be credible [28].

The pandemic forced a more efficient adoption of telemedicine services, and the telehealth tools increased the availability of more personalized medical services [40]. The search for safer alternatives for the patient and medical staff to the traditional medical services easily found telemedicine as a response to the COVID-19 pandemic. The implemented solutions focused on the discussed primary care telemedicine and all of the medical specialties, including telerehabilitation and telemonitoring [41].

Teleconsultation is an example of a telemedicine solution of communication at a distance in healthcare, focused on diagnosing disorders [42], and as a part of telerehabilitation systems in posthospital patient care [15].

In March 2020, the state of the epidemic was announced in Poland. In response to the epidemic, restrictions were gradually introduced to reduce coronavirus transmission. During a routine visit, the patients waiting for an appointment in the waiting room or during transport to a healthcare facility could expose themselves, other patients, or healthcare personnel to infections [43,44]. In August 2020, the regulations for the PCT standards were published. In November 2020, the National Health Fund announced medical teleconsultations in primary care clinics to serve patients during the coronavirus pandemic. A list of the primary care facilities in all of the voivodeships was also announced. In January 2021, the first stage of vaccination against COVID-19 began. People over 60 years of age were the first group of patients who had the opportunity to be vaccinated against COVID-19. From March 2021, new rules for using medical visits in clinics were introduced. A primary care physician could not refuse a face-to-face visit to a patient who refuses PCT and has no suspicion of COVID-19 infection [45].

Nevertheless, in 2021, the percentage of seniors using the services of PCT was still the highest, and significantly increased compared to the previous year. In response to the increased number of coronavirus infections, another lockdown was introduced in March 2021, which increased the number of served medical teleconsultations (Table 1, Figure 1). The number of patients accessing teleconsultations increased with age, resulting from an effective educational campaign addressed to seniors (they were asked to limit their activity related to leaving home and interpersonal contacts) (Table 2). The benefits of introducing telemedicine services are noticeable, not only in the case of considering telemedicine as a way of counteracting the spread of SARS-CoV-2. This is evidenced by other countries’ experience, where telemedicine developed well before the outbreak of SARS-CoV-2. Finland is an example of such a country. By introducing e-health services in Finland, patients received more personalized healthcare. Through e-consultations, patients living in regions with difficult access have the opportunity to use the services, which contributes to a faster diagnosis [30].

Along with telemedicine services, time and cost savings, and improvements in treatment were observed. By introducing the “e-prescription” monitoring system, the number of overused drugs, inadequate ways of consuming them, and combining them with other medications decreased, increasing patient safety [30,46,47,48]. According to the European Observatory on Health Systems and Policies, in response to COVID-19, telemedicine solutions have also been implemented in other European countries [30]. In Croatia, in the first 12 months after the announcement of the COVID-19 pandemic, it was registered that 42% of the population was provided with a teleconsultation, which was 3% more than the European Union average. The number of medical teleconsultations in Denmark has similarly increased in response to the COVID-19 pandemic. Medical teleconsultations increased the total number of consultations by 9% in 2020 compared with 2019, despite reduced face-to-face appointments. In Finland, in January–February 2020, the number of appointments with primary care physicians was 6.8 million and decreased by 1 million in April–May 2020. In the following months, the number of medical consultations exceeded eight million per month, and the number of medical teleconsultations increased from 0.1 million to 1.2 million on average per month in 2020. In France, several measures were taken to increase access to telemedicine services. The number of medical consultations and teleconsultations reached one million per week in April 2020, and, compared with the previous month, this number increased by 90%. The cost of the telemedicine services was covered entirely by the Health Insurance System. In Germany, it was reported that 23% of citizens were provided with a teleconsultation during the first 12 months of the pandemic. In Slovenia, 96% of e-prescriptions were issued in 2020, and about 65% of the citizens were supplied with a teleconsultation within 12 months [30].

### Strengths and Limitations

The study was conducted based on statistical data from the National Health Fund, including public health data for Poland. The dataset is limited to 2020 and 2021, due to the duration of the pandemic. The study failed to assess the scope of PCT and the entirety of the services offered to patients, whether there were only simple e-prescriptions or more sophisticated techniques used for medical services. The study was limited to primary care, so other specialties were not considered. However, this analysis will be an appropriate topic for the following research.

The research’s undisputed advantage is the study’s scale, which covers over 38 million citizens of the country. To the authors, no other studies have been conducted on this scale. An additional limitation of the conducted research is taking into account only data from the public sector, which does not constitute 100% coverage of medical services. Obtaining data from the private and subscription services industry, which have recently gained importance, could, to some extent, reduce the bias of the analysis carried out.

Due to the reporting period, the data were unavailable, and so the year 2022 was not included in the study.

## 5. Conclusions

The data analysis shows that COVID-19 provided an impulse for developing telemedicine in primary care. Before the pandemic, the only services within primary care that used telemedicine tools were consultations between medical professionals. Introducing recommendations to minimize the exposure to COVID-19 meant that beneficiaries began to use teleconsultation provided as part of primary care. COVID-19, along with the presented recommendations, was an impulse to minimize barriers, not only legal but also awareness. Due to the introduced legal regulations, patients used direct visits only when they were necessary, due to the patient’s health condition. The high percentage of PCTs administered to older patients proves the reduction in the telemedicine awareness barrier. The most common beneficiaries of PCTs carried out in 2020 and 2021 were patients aged 70 and older.

Healthcare services provided remotely or with remote communication devices are becoming part of medical services and are more and more often used by medical professionals and patients. The healthcare industry has experienced a change in how medical services are provided during the COVID-19 pandemic. The benefits resulting from implementing projects and telemedicine solutions are visible in the highly developed and developing countries. Research supports the idea that telemedicine is at least as good as conventional services, in terms of effectiveness, cost, and patient outcomes [25,49,50,51,52,53,54,55,56,57,58,59,60]. The use of telemedicine tools as an alternative to traditional healthcare services has positively influenced the participants in the healthcare sector. The skillful use of telemedicine solutions can increase the efficiency and quality of medical services provided, through faster diagnosis, patient education about their health, and the number of potential patients. Teleconsultations increase epidemiological safety, and significantly reduce the exposure of patients and healthcare personnel to the direct transmission of a virus vector and the spread of infection.

By introducing recommendations to minimize the spread of the SARS-CoV-2 virus, the interest in using telemedicine services worldwide increased, and telemedicine has become a way of using healthcare services.

The attempt to summarize the factors influencing the successful implementation of PCTs indicates three important factors: 1. reduction in barriers in telemedicine (seen as increasing use of PCT with age); 2. reimbursement (based on published regulations [38]; and 3. avoiding the risk of contact and infection with a highly infectious pathogen [61,62].

Currently, PCTs are still continuing to be provided to patients, and are an excellent addition to the medical services provided. Initial distance interviews with triage elements are used to assess the need for the patient’s physical presence during the consultation. The development of research methods, medical knowledge, and new technologies make it possible to increase the possibility of a safe and reliable medical examination at a distance, at the patient’s home, without needing a direct visit.

Future research should focus on PCTs’ quality, implementation of new tools supporting tele-diagnostics and tele-treatment, the satisfaction of both doctors and patients participating in telemedicine consultations, and necessary patient-reported outcomes.

## Figures and Tables

**Table 1:** The summary table of the number of residents of voivodeships and the incidence of PCT per 10,000 residents/year.

Name of the National Health Fund Regional Branch	Number of Residents		The PCT Incidence/10,000 Residents		Increase of the PCT per National Health Fund Regional Branch (%)
	2020	2021	2020	2021	
Dolnośląski	2,900,163	2,891,321	3299.22	10,681.39	323.8
Kujawsko-Pomorski	2,072,373	2,061,942	3289.73	10,026.96	304.8
Lubelski	2,108,270	2,095,258	1844.61	9856.08	534.3
Lubuski	1,011,592	1,007,145	2103.17	7540.91	358.5
Łódzki	2,454,779	2,437,970	3464.65	10,460.21	301.9
Małopolski	3,410,901	3,410,441	2805.13	8783.99	313.1
Mazowiecki	5,423,168	5,425,028	3785.60	10,718.28	283.1
Opolski	982,626	976,774	3228.91	9523.29	294.9
Podkarpacki	2,127,164	2,121,229	1668.08	7331.97	439.5
Podlaski	1,178,353	1,173,286	1496.44	7761.24	518.6
Pomorski	2,343,928	2,346,671	3114.57	10,712.22	343.9
Śląski	4,517,635	4,492,330	2603.38	10,492.96	403.1
Świętokrzyski	1,233,961	1,224,626	3127.25	9630.50	308
Warmińsko mazurski	1,422,737	1,416,495	2031.49	8052.34	396.4
Wielkopolski	3,498,733	3,496,450	2633.84	8139.02	309
Zachodniopomorski	1,696,193	1,688,047	3959.53	11,084.99	280

**Table 2:** The table shows the numbers of patients served with a PCT in 2020 and 2021 with age groups and the population size per 10,000 residents.

Age Group	Population 2020	Population 2021	2020	2021	PCTs 2020	PCTs 2021	Rate 2020	Rate 2021
0–4	1,911,494	1,876,822	6.09%	5.7%	677,058	2,104,353	3542.04	11,212.32
5–9	1,930,096	1,905,991	3.18%	3.5%	353,729	1,298,516	1832.70	6812.81
10–14	2,042,479	2,072,944	2.38%	2.5%	264,719	931,171	1296.07	4492.02
15–19	1,798,052	1,802,588	2.25%	2.4%	250,354	870,700	1392.36	4830.28
20–24	1,999,667	1,940,927	2.92%	2.9%	325,281	1,090,852	1626.68	5620.26
25–29	2,457,738	2,348,271	3.82%	3.7%	425,276	1,351,488	1730.36	5755.25
30–34	2,867,784	2,784,304	4.65%	4.5%	517,493	1,651,719	1804.50	5932.25
35–39	3,228,463	3,211,368	5.85%	5.7%	650,720	2,091,457	2015.57	6512.67
40–44	3,054,544	3,079,795	6.35%	6.1%	706,426	2,254,233	2312.71	7319.43
45–49	2,646,756	2,742,437	6.33%	6.2%	704,608	2,286,187	2662.16	8336.33
50–54	2,275,278	2,299,098	6.29%	6.1%	699,145	2,255,743	3072.79	9811.42
55–59	2,363,518	2,294,188	7.23%	7.0%	804,709	2,586,696	3404.71	11,275.00
60–64	2,719,848	2,628,254	9.31%	9.2%	1,035,921	3,420,617	3808.75	13,014.79
65–69	2,487,083	2,509,175	10.03%	10.3%	1,115,851	3,798,019	4486.59	15,136.52
>70	4,571,373	4,666,062	23.30%	24.4%	2,591,857	9,009,907	5669.76	19,309.44
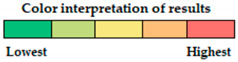								

**Table 3:** The table presents the Pearson correlation coefficient results between the average/median PIT/CIT and the incidence of PCTs per 10,000 residents.

Voivodeship	The Median Age in a Voivodeship 2020	The Average Amount of PIT 2019	The Average Amount of CIT 2019	The Median Amount of PIT 2019	The Median Amount of CIT 2019	The Incidence of PCTs per 10,000 Residents in 2020	The Incidence of PCTs per 10,000 Residents in 2021
Dolnośląskie	41.7	3608	167,128	3385	60,960	3299.22	10,681.39
Kujawsko-Pomorskie	42.3	2860	164,415	2655	86,730	3289.73	10,026.96
Lubelskie	41.7	2771	128,039	2501	92,028	1844.61	9856.08
Lubuskie	41.8	3122	66,918	3032	69,496	2103.17	7540.91
Łódzkie	41.6	3078	162,752	2844	128,357	3464.65	10,460.21
Małopolskie	43.3	3145	126,988	2953	118,355	2805.13	8783.99
Mazowieckie	40.4	3603	278,619	3008	128,451	3785.60	10,718.28
Opolskie	41.2	3204	129,224	3104	101,827	3228.91	9523.29
Podkarpackie	43.4	2818	130,746	2692	129,140	1668.08	7331.97
Podlaskie	40.7	2711	161,479	2612	120,793	1496.44	7761.24
Pomorskie	41.8	3526	118,704	3167	82,414	114.57	10,712.22
Śląskie	40.3	3890	143,754	3977	107,455	2603.38	10,492.96
Świętokrzyskie	43.0	2756	261,533	2752	173,287	3127.25	9630.50
Warmińsko-Mazurskie	43.0	2855	53,286	2716	36,408	2031.49	8052.34
Wielkopolskie	41.2	3065	193,677	2894	14,269	2633.84	8139.02
Zachodniopomorskie	40.5	3143	63,575	3023	46,432	3959.53	11,084.99
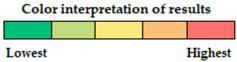							

## Data Availability

The data presented in this study are available on request from the corresponding author.
